# ID 60 - Preditores do Tempo de Internação e Custos Hospitalares para Pacientes Submetidos a Transplante de Célula-Tronco Hematopoiética em um Hospital Público Brasileiro

**DOI:** 10.5327/2237-9622.2025.v34s1.60

**Published:** 2025-11-25

**Authors:** Anna Ludovico Stollenwerk, Anna Ludovico Stollenwerk, Roselene Mesquita Augusto Passos, Laís Maria de Campos, Deyvid Fernando Mattei da Silva, Leila de Lurdes Martins Perobelli

**Keywords:** doenças hematológicas, enfermedades hematológicas, *hematologic diseases*, transplante de medula óssea, trasplante de médula ósea, *bone marrow transplantation*, tempo de internação, *tiempo de internación*, *length of stay*, custos e análise de custo, cos

## Abstract

**Introdução:**

O transplante de células-tronco hematopoiéticas (TCTH) é essencial para tratar doenças hematológicas, mas envolve altos custos, desafiando sistemas de saúde com recursos limitados. Fatores como o tipo de transplante, internação e complicações impactam significativamente os custos. Este estudo tem como objetivo analisar os preditores do tempo de internação e os custos agregados hospitalares associados ao TCTH em um hospital público brasileiro, contribuindo para a formulação de políticas públicas que promovam eficiência e equidade no acesso ao tratamento.

**Método:**

Estudo retrospectivo que analisou 231 prontuários de pacientes submetidos a TCTH autólogo ou alogênico aparentado, em um hospital público de São Paulo, entre 2017 e 2020. As variáveis do estudo foram: idade, gênero, diagnóstico, escore de risco, tipo de TCTH, fonte de células-tronco, protocolo de condicionamento, tempo de internação, complicações, necessidade de UTI e custos hospitalares agregados por meio do método de macrocusteio.(3) A análise estatística foi realizada com R 4.1.0 e Jamovi.

**Resultados:**

O tempo de internação variou de 14 a 81 dias (mediana=23). Fatores que prolongaram significativamente o tempo de internação (p <0,05) incluíram menor faixa etária, diagnóstico de leucemia mieloide aguda e linfoma de Hodgkin, escore de risco muito alto pelo (DRI), TCTHs alogênicos haploidênticos, intensidade de condicionamento reduzido e presença de complicações. O tempo de aplasia também esteve significativamente associado ao tempo total de internação (p < 0,001). Cerca de 90% dos TCTHs realizados apresentaram alguma complicação. A principal foi a ocorrência de infecção, incluindo neutropenia febril (30,3%), infecção documentada (52,8%) e sepse (10,8%). Das infecções documentadas, a mais frequente foi pneumonia (15,6%) e infecção de corrente sanguínea relacionada a cateter (12,6%). Outras complicações frequentes foram mucosite (52%) e necessidade de nutrição parenteral (10,8%). Apenas 3% evoluíram a óbito em decorrência de infecção grave. O custo do TCTH foi estimado a partir da duração da hospitalização e variaram significativamente conforme o tipo de transplante e a ocorrência de complicações. No transplante autólogo, a mediana de dias de internação foi de 17 dias, com um custo de R$ 81.442,24 sem complicações, aumentando para 22 dias e R$ 105.395,84 com complicações, e chegando a 33 dias e R$ 168.390,73 quando o paciente precisou de UTI. No transplante alogênico, o tempo de internação foi de 25 dias, com um custo de R$ 119.768,00 sem complicações, aumentando para 30 dias e R$ 143.721,60 com complicações, e alcançando 37 dias e R$ 197.987,53 em casos que necessitaram de UTI.

**Conclusão:**

Este estudo oferece uma visão abrangente dos preditores de tempo de internação e custos associados ao TCTH, utilizando uma amostra maior que outros estudos brasileiros. Os custos agregados foram menores que os reportados nos EUA, evidenciando variações entre países. A análise das complicações pós-TCTH e seu impacto nos custos destaca a importância de estratégias eficazes de prevenção e manejo. Os resultados contribuem para o entendimento dos fatores que afetam o tempo de internação e custos no Brasil, com implicações para políticas públicas e planejamento dos serviços de TCTH.

